# AdaBoost-based multiple SVM-RFE for classification of mammograms in DDSM

**DOI:** 10.1186/1472-6947-9-S1-S1

**Published:** 2009-11-03

**Authors:** Sejong Yoon, Saejoon Kim

**Affiliations:** 1 Department of Computer Science and Engineering, Sogang University,1 Shinsu-dong, Mapo-gu, Seoul, Korea

## Abstract

**Background:**

Digital mammography is one of the most promising options to diagnose breast cancer which is the most common cancer in women. However, its effectiveness is enfeebled due to the difficulty in distinguishing actual cancer lesions from benign abnormalities, which results in unnecessary biopsy referrals. To overcome this issue, computer aided diagnosis (CADx) using machine learning techniques have been studied worldwide. Since this is a classification problem and the number of features obtainable from a mammogram image is infinite, a feature selection method that is tailored for use in the CADx systems is needed.

**Methods:**

We propose a feature selection method based on multiple support vector machine recursive feature elimination (MSVM-RFE). We compared our method with four previously proposed feature selection methods which use support vector machine as the base classifier. Experiments were performed on lesions extracted from the Digital Database of Screening Mammography, the largest public digital mammography database available. We measured average accuracy over 5-fold cross validation on the 8 datasets we extracted.

**Results:**

Selecting from 8 features, conventional algorithms like SVM-RFE and multiple SVM-RFE showed slightly better performance than others. However, when selecting from 22 features, our proposed modified multiple SVM-RFE using boosting outperformed or was at least competitive to all others.

**Conclusion:**

Our modified method may be a possible alternative to SVM-RFE or the original MSVM-RFE in many cases of interest. In the future, we need a specific method to effectively combine models trained during the feature selection process and a way to combine feature subsets generated from individual SVM-RFE instances.

## Background

Applications of artificial intelligence and machine learning techniques in medicine are now common and computer aided diagnosis (CADx) systems are one of those successful applications. Breast cancer, the most common cancer in women and second largest cause of death [1], is the disease which CADx systems are expected to be employed most successfully. To apply CADx systems, various imaging methods are available to reflect the inside tissue structure of breasts. Digital mammography using low-dose x-ray is one of those methods and is the most popular one worldwide. It has advantages over other methods such as sonar or magnetic resonance imaging (MRI) due to low cost and wide availability [2]. With digital mammography devices, doctors are able to find abnormal lesions which cannot be recognized using clinical palpation on breasts. CADx systems are applied on those images to detect and diagnose abnormalities. Since the early detection of breast cancer is important to ensure successful treatment of the disease, recent advances in research community have concentrated on improving the performance of CADx systems. Improvements in CADx systems can be obtained by solving two classification tasks: (1) detect more abnormalities or (2) distinguish actual malignant cancers from benign ones. Detecting abnormalities from a digitized mammogram is a relatively easy task and many improvements have been achieved while the latter is still a major area of research [3]. To achieve better performance, both classic and modern machine learning approaches such as Bayesian networks [4], artificial neural networks [5,6] and support vector machines (SVMs) [5,7] have been applied. However, the performance of CADx systems is still not as high as required for practical usage. This problem can be partially solved by using a better feature selection method that optimally fits to the mammogram classification problem [3].

We propose a new feature selection method for SVMs in this paper. Our method is based on SVM-Recursive Feature Elimination (SVM-RFE) [8] and its ensemble variant Multiple SVM-RFE [9]. We have conducted a comparison of the classification performance with baseline methods and two other SVM-RFE based feature selection methods, JOIN and ENSEMBLE, proposed by other groups [10]. To compare performances of methods, we prepared a dataset consisting of mass and calcification lesions extracted from Digital Database of Screening Mammography (DDSM) [11], the largest publicly available mammogram database.

## Methods

### Notations

Let us suppose that a data set consists of *N *examples **x**_1_,..., **x**_*N *_each of which has *P *features {1,..., *P*}.

Let **x**_*n *_= (*x*_1, *n*_,..., *x*_*P*, *n*_) be the *n*-th example where *n *∈ {1,..., *N*}, and the *i*-th feature value, *i *∈ {1,..., *P*}, of the *n*-th example is denoted by *x*_*i*, *n*_. Class labels of the *N *examples will be denoted by **y **= (*y*_1_,..., *y*_*N*_).

In this paper, we only consider a binary classification problem because we are interested in distinguishing benign and malignant examples. Overall, the labeled data set is expressed as {(**x**_1_, *y*_1_),..., (**x**_*N*_, *y*_*N*_)}.

### SVM

SVM is one of the most popular modern classification methods. Based on the structural risk minimization principal, SVM defines an optimal hyperplane between samples of different class labels. The position of the hyperplane is adjusted so that the distance from the hyperplane to a nearest sample, or margin, is maximized.

Moreover, if the SVM cannot define any hyperplane that separates examples in linear space, it can use kernel functions to send examples to any kernel space where the hyperplane can separate examples. Although we can use any kernel function meeting Mercer's Theorem for SVM, we consider widely-used the linear and Gaussian radial basis function (RBF) kernels only in this research.

### SVM-RFE

SVM is a powerful classification method but it has no feature selection method. Therefore, a wrapper-type feature selection method, SVM-RFE, was introduced [8]. SVM-RFE generates ranking of features by computing information gain during iterative backward feature elimination. The idea of information gain computation is based on Optimal Brain Damage (OBD) [12]. In every iterative step, SVM-RFE sorts the features in working set in the order of difference of the obejective functions and removes a feature with the minimum difference. Defining *IG*(*k*) as information gain when *k*-th feature is removed, overall iterative algorithm of SVM-RFE is shown in Algorithm 1.

### ENSEMBLE and JOIN

SVM-RFE [8] has two parameters that need to be determined. The first parameter decides how many features should be used to obtain best performance. The second parameter specifies what portion of features should be eliminated in each iteration. To resolve this issue, a simple approach can be easily

**Algorithm 1 **SVM-RFE

**Require**: Feature lists *R *= [] and *S *= [1,..., *P*]

1:   **while ***S *≠ [] **do**

2:      Train a SVM with features in *S*

3:      **for all ***k*-th feature in *S ***do**

4:         Compute *IG*(*k*)

5:      **end for**

6:      *e *= arg min_*k*_(*IG*(*k*))

7:      *R *= [*e*, *R*]

8:      *S *= *S *- [*e*]

9:   **end while**

10:   **return ***R*

implemented. First, we separate given training set into a partial training set and a hold-out set. Then, we apply Algorithm 2 with some parameter 'threshold'.

Score of each feature subset *R*_*o *_is computed as



where *err*(*R*_*o*_) is the error of SVM trained using *R*_*o *_and tested with hold-out set. Using this method, we can obtain a feature subset *R *which yields reasonably small amount of error on trained dataset. Utilizing this algorithm as base, Jong et al. [10] proposed two methods, ENSEMBLE and JOIN to combine multiple rankings generated by SVM-RFE as in Algorithm 3 and 4.

In this paper, we used 25% of training set as hold-out set and used same sets of thresholds and cutoffs as in [10], i.e., {0.2, 0.3, 0.4, 0.5, 0.6, 0.7} and {1, 2, 3, 4, 5}.

**Algorithm 2 **SVM-RFE(threshold)

**Require**: Ranked feature lists *R *= [], *R*_*i *_= [] where *i *= 1,..., *P *and *S' *= [1,..., *P*]

1:   i = 1

2:   **while ***S' *≠ [] **do**

3:      Train an SVMs using a partial trainset with features in *S'*

4:      **for all **features in *S' ***do**

5:         Compute ranking of features as in SVM-RFE

6:      **end for**

7:      *R*_*i *_= *S'*

8:      Eliminate threshold percent of lesser-important features from *S'*

9:      *i *= *i *+ 1

10:   **end while**

11:   *R *= *R*_*o *_where *R*_*o *_yields minimum score on hold-out set.

12:   **return ***R*

**Algorithm 3 **ENSEMBLE(*v*_1_, *v*_2_,.., *v*_*k*_)

1:   **for **threshold *v *∈ {*v*_1_, *v*_2_,..., *v*_*k*_} do

2:      *R*_*v *_= SVM-RFE(*v*)

3:   **end for**

4:   **return **a majority vote classifier using SVMs trained by .

**Algorithm 4 **JOIN(cutoff, *v*_1_, *v*_2_,..., *v*_*k*_)

1:   **for **threshold *v *∈ {*v*_1_, *v*_2_,..., *v*_*k*_} **do**

2:      *R*_*v *_= SVM-RFE(*v*)

3:   **end for**

4:   *R *= features selected at least cutoff times in {}

5:   **return **a SVM trained with *R*

### Multiple SVM-RFE with bootstrap

Multiple SVM-RFE (MSVM-RFE) [9] is a recently introduced SVM-RFE-based feature selection algorithm. It exploits an ensemble of SVM classifiers and cross validation schemes to rank features. First, we make *T *subsamples from the original training set. Then, supposing that we have *T *SVMs trained using different subsamples, we calculate the corresponding discriminant information gain associated with each feature of each SVM. To compute this information gain, we use the same method as in SVM-RFE [8]. Exploiting the objective function of SVM, and its Lagrangian solution *λ*, we can derive a cost function



where **H **is a matrix with elements *y*_*q*_*y*_*r*_*K*(**x**_*q*_, **x**_*r*_) and **1 **is a *N *dimensional vector of ones while *K*(·) is a kernel function and 1 ≤ *q*, *r *≤ *N*. Since we are looking for the subset of features that has the best discriminating power between classes, we compute the difference in cost function for each elimination of *i*-th input feature, leaving Lagrangian multipliers unchanged. Therefore, the ranking for the *i*-th feature of *j*-th SVM can be defined as



where **H**(-*i*) denotes that *i*-th feature was removed from all elements in **H**. Then, considering *DJ*_*j *_as a weight vector of features for *j*-th SVM, we normalize all *T *weight vectors such as *DJ*_*j *_= *DJ*_*j*_/||*DJ*_*j*_||. This gives us *T *weight vectors each with *P *elements. Here, each element in the vector stands for a information gain achieved by eliminating the corresponding feature. After normalizing weight vectors for each SVM, we can compute each feature's ranking score



with *μ*_*i *_and *σ*_*i *_defined as:





The algorithm then applies this method to the training set with *k*-fold cross validation scheme. If we perform 5-fold cross validation and generate 20 subsamples in each fold, we will eventually have *T *= 100 SVMs to combine. The overall MSVM-RFE algorithm is described in Algorithm 5.

**Algorithm 5 **MSVM-RFE

**Require**: Ranked feature lists *R *= [] and *S' *= [1,..., *P*]

1:   **while ***S' *≠ [] **do**

2:      Train *T *SVMs using *T *subsamples with features in *S'*

3:      **for all ***j*-th SVM 1 ≤ *j *≤ *T ***do**

4:         **for all ***i*-th feature 1 ≤ *i *≤ *P ***do**

5:            Compute *DJ*_*ji*_

6:         **end for**

7:         Compute *DJ*_*j *_= *DJ*_*j*_/||*DJ*_*j*_||

8:      **end for**

9:      **for all **feature *l *∈ *S' ***do**

10:         Compute *c*_*l *_using Equation (1)

11:      **end for**

12:      *e *= arg min_*l*_(*c*(*l*)) where *l *∈ *S'*

13:      *R *= [*e*, *R*]

14:      *S' *= *S' *- [*e*]

15:   **end while**

16:   **return ***R*

One should note that original MSVM-RFE proposed in [9] uses cross-validation scheme when generating subsamples. However, we omitted this step because combining boosting into the original MSVM-RFE algorithm with cross-validation scheme is very complex and may confuse the purpose of this study.

### Multiple SVM-RFE with boosting

When making subsamples, original MSVM-RFE uses the bootstrap approach [13]. This ensemble approach builds replicates of the original data set *S *by random re-sampling from *S*, but with replacement *N *times, where *N *is the number of examples. Therefore, each example (**x**_*n*_, *y*_*n*_) may appear more than once or not at all in a particular replicate subsample. Statistically, it is desirable to make every replicate differ as much as possible to gain higher improvement of the ensemble. The concept is both intuitively reasonable and theoretically correct. However, as the architecture of MSVM-RFE uses simple bootstrapping, it naturally follows that utilizing another popular ensemble method, boosting [14], instead of bootstrapping for two reasons. First, boosting outperforms bootstrapping on average [15,16], and secondly, boosting of SVMs generally yields better classification accuracy than bootstrap counterpart [17]. Therefore, to make use of ensemble of SVMs effectively, it may be worthwhile to use boosting instead of bootstrapping. For this reason, we applied AdaBoost [14], a classic boosting algorithm, to MSVM-RFE algorithm instead of bootstrapping in this work.

Unlike the simple bootstrap approach, AdaBoost maintains weights of each example in *S*. Initially, we assign same value of weight to *n*-th example *D*_1_(*n*) = 1/*N *where 1 ≤ *n *≤ *N*. Each iterative process consists of four steps. At first, the algorithm generates a bootstrap subsample according to weight distribution at *t*-th iteration *D*_*t*_. Next, it trains an SVM using the subsample. Third, it calculate the error using the original example set *S*. Finally it updates the weight value so that the probability of correctly classified examples is decreased while that of incorrect ones is increased. This update procedure makes next bootstrap pick more incorrectly classified examples, i.e. difficult-to-classify examples than easy-to-classify ones. The iterative re-sampling procedure MAKE_SUBSAMPLES() using AdaBoost algorithm is described in Algorithm 6.

**Algorithm 6 **MAKE_SUBSAMPLE

**Require**: *S *= {(**x**_*n*_, *y*_*n*_)}, *D*_1_(*n*) = 1/*N*, *n *= 1,..., *N*;

1:   **for ***j *= 1 to *T ***do**

2:      Build a bootstrap *B*_*j *_= {(**x**_*n*_, *y*_*n*_)|*n *= 1,..., *N*} based on weight distribution *D*_*j*_

3:      Train a SVM hypothesis *h*_*j *_using *B*_*j*_

4:      

5:      **if **ϵ_*j*_≥ 0.5 **then**

6:         Goto line 2

7:      **end if**

8:      *α*_*j *_= (1/2)*ln*((1 - ϵ_*j*_)/ϵ_*j*_), *α*_*j *_∈ **R**

9:      *D*_*j*+1_(*n*) = (*D*_*j*_(*n*)/*Z*_*j*_) × exp(-*α*_*j*_*y*_*n*_*h*_*j*_(**x**_*n*_)) where *Z*_*j *_is a normalization factor chosen so that *D*_*j*+1 _also be a probability distribution

10:   **end for**

11:   **return ***B*_*j*_, *α*_*j *_where 1 ≤ *j *≤ *T*

In addition to modifying re-sampling method, we made a change in ranking criterion of original MSVM-RFE. In this MSVM-RFE with Boosting method, the weight vector *DJ*_*j *_of *j*-th SVM undergoes one more process between normalization and feature ranking score calculation. Since the contribution of each SVM in ensemble to the overall classification accuracy is unique, we multiply another weight factor to the normalized feature weight vector *DJ*_*j*_. The new weight factor is obtained from the weight of hypothesis classifier calculated during the re-sampling process of AdaBoost. By multiplying this weight *α*_*j *_to *DJ*_*j*_, we can grade the overall feature weight more coherently. The overall iterative algorithm of MSVM-RFE with AdaBoost is described in Algorithm 7.

**Algorithm 7 **MSVM-RFE with AdaBoost

**Require**: Ranked feature lists *R *= [] and *S'*= [1,..., *P*]

1:   MAKE_SUBSAMPLES(*B*_*t*_, *α*_*t*_); *t *= 1,..., *T*

2:   **while ***S' *≠ [] **do**

3:      Train *T *SVMs using *B*_*t*_, with features in set *S'*

4:      Compute and normalize *T *weight vectors *DJ*_*j *_as in MSVM-RFE where 1 ≤ *j *≤ *T*

5:      **for ***j *= 1 to *T ***do**

6:         *DJ*_*j *_= *DJ*_*j *_× *ln*(*α*_*j*_)

7:      **end for**

8:      **for all **feature *l *∈ *S' ***do**

9:         Compute the ranking score *c*_*l *_using Eq. (1)

10:      **end for**

11:      *e *= *argmin*_*l*_(*c*_*l*_) where *l *∈ *S'*

12:      *R *= [*e*, *R*]

13:      *S' *= *S' *- [*e*]

14:   **end while**

15:   **return ***R*

Note that we took logarithm of hypothesis weights instead of raw values in order to avoid radical changes in ranking criterion. Since boosting algorithm overfits by nature and SVM, the base classifier, is relatively strong classifier, the error rate of hypothesis increases drastically as iteration in MAKE_SUBSAMPLES() progresses. We have witnessed this overfitting problem by preliminary experiment and solved the problem by taking logarithm to the hypothesis weight. Computation time of MSVM-RFE with boosting can also be explained here. From our experiments, we found that there is no significant difference between the original MSVM-RFE and MSVM-RFE with boosting as the number of subsamples generated by MAKE_SUBSAMPLES() decreases.

Lastly, unlike the conventional boosting algorithm application, we only exploit bootstrap subsamples generated by the algorithm and dismiss trained SVMs for the following reasons:

• We are primarily interested in feature ranking and not the aggregation of weak hypotheses.

• Since we are using SVM-RFE for eventual classification method, this require a certain criterion to pick appropriate number of features from different boosted models.

In preliminary experiments using same number of features and simple majority-voting aggregation, SVM-RFE using boosted models did not show significance in accuracy improvement. However, we could find some evidences that ensemble of SVMs can be useful in mammogram classification.

## Results

In this section, we first describe dataset, features and experimental framework we used. Then we draw results of the experiments including analysis on them.

### Dataset

The DDSM database provides about 2500 mammogram cases that were gathered from 1988 to 1999. Four U.S. medical institutions offered the data to construct DDSM. This includes Massachusetts General Hospital (MGH), Wake Forest University School of Medicine (WFUSM), Sacred Heart Hospital (SHH) and Washington University in St. Louis (WU). All mammogram cases we used in this paper contain one or more abnormalities which can be classified into benign or malignant group following their biopsy results. Table 1 summarizes the statistics of abnormalities from each digitizer type and institution.

**Table 1:** Dataset Information

**institution**	**mass**		**calcification**	
	**benign**	**malignant**	**benign**	**malignant**
MGH	482	365	381	323
WU	154	115	41	98
WFUSM	163	255	188	159
SHH	324	380	207	140

total	1123	1115	817	720

MGH = Massachussetts General Hospital; WU = Washington University at Saint Louis; WFUSM = Wake Forest University School of Medicine; SHH = Sacred Heart Hospital

Mammogram data from DDSM were gathered and preprocessed through the following steps. First, we extracted meta information from text file in the database. These features are based on Breast Imaging Reporting and Data System (BI-RADS) introduced by the American College of Radiology [18]. Table 2 summarizes these encoded features. We employed a rank ordering system proposed by other group when encoding these features [19]. Next, we computed statistical features that are popular in image processing community. The statistical features are computed using intensity level of pixels in the region of interest in each case. We used same features which are used in another study [6] and the exact formulas are described in [20]. We also normalized these statistical features after extracting because their raw values were too big compared to BI-RADS features and to facilitate SVM to train efficiently with respect to time.

**Table 2:** BI-RADS mammographic features

**feature type**	**description or numeric value**
mass shape	no mass(0), round(1), oval(2), lobulated(3), irregular(4)
mass margin	no mass(0), well circumscribed(1), microlobulated(2), obscured(3), ill-defined(4), spiculated(5)
calcification type	no calc.(0), milk of calcium-like(1), eggshell(2), skin(3), vascular(4), spherical(5), suture(6), coarse(7), large rod-like(8), round(9), dystrophic(10), punctate(11), indistinct(12), pleomorphic(13), fine branching(14)
calcification distribution	no calc.(0), diffuse(1), regional(2), segmental(3), linear(4), clustered(5)
density	1, 2, 3, 4
assessment	1, 2, 3, 4, 5

density: 1 = sparser, 4 = denser;

### Performance comparison

In sum, we prepared a total of 16 datasets each with 8 and 22 features, from each mass and calcification lesion of each institution. All SVM-RFE based methods are tested using 5-fold cross validation on each dataset. We computed area under Receiver Operating Characteristic (ROC) curves (*A*_*z*_) using the output of SVMs and feature ranking produced by each method.

Before comparing the methods explained in the previous section, we did some preliminary experiments comparing different kernels and parameters to find optimal kernel and parameters. The result of this experiment is summarized in Table 3 and Table 4. We used the best-performing parameter and kernel (radial basis function, or RBF) from this experiment of this study.

**Table 3:** Comparison of kernels in terms of maximum Az value of mass dataset

**kernel type**	**MGH**		**WU**		**WUFSM**		**SHH**	
	**8**	**22**	**8**	**22**	**8**	**22**	**8**	**22**
linear	0.90391	**0.90364**	0.94571	0.92159	0.85718	0.87159	0.97150	**0.97036**
RBF	**0.96664**	0.88597	**0.95955**	**0.92540**	**0.91906**	**0.91671**	**0.97404**	0.95716
*C*	10	5	10	10	10	10	10	10
*γ*	0.25	0.06	0.5	0.075	0.15	0.1	0.5	0.05

Same tradeoff parameter value *C *is used for both linear and RBF kernels.

**Table 4:** Comparison of kernels in terms of maximum Az value of calcification dataset

**kernel type**	**MGH**		**WU**		**WUFSM**		**SHH**	
	**8**	**22**	**8**	**22**	**8**	**22**	**8**	**22**
linear	0.72686	0.72625	0.89981	**0.90870**	0.74046	0.77509	0.89603	0.92705
RBF	**0.91042**	**0.76826**	**0.99192**	0.88155	**0.93625**	**0.89079**	**0.96280**	**0.94826**
*C*	1	10	1	5	10	20	10	10
*γ*	1.5	0.1	1	0.05	0.4	0.05	0.15	0.05

Same tradeoff parameter value *C *is used for both linear and RBF kernels.

The overall performance comparison result is summarized from Table 5 through Table 8. Note that numbers in parenthesis of JOIN methods are cutoff values used. Analyzing the result, it is clear that the MSVM-RFE based methods outperforms baseline classifiers, SVM and other SVM-RFE feature selection methods, ENSEMBLE and JOIN in the majority of cases although SVM-RFE dominated in 4 out of 16 datasets. Comparing the two MSVM-RFE based algorithms, we could find that MSVM-RFE with boosting can achieve better or at least competitive performance especially in datasets with 22 features. In 3 out of 4 mass datasets, MSVM-RFE with boosting outperformed any other methods under consideration. Although the original MSVM-RFE method yielded the best performance in 3 out of 4 calcification datasets, we think the MSVM-RFE with boosting has yet more margin to be improved as we already mentioned in the previous chapter. Any method that can effectively exploit the trained SVMs during feature selection progress may be the future key improvement for MSVM-RFE with boosting.

**Table 5:** Comparison of methods by maximum Az value using 8 features (Mass)

	***T***	**MGH**	**WU**	**WFUSM**	**SHH**
SVM		0.95821	0.97247	0.92252	0.97401
SVM-RFE		**0.96218**	0.97734	0.92252	0.97401
					
ENSEMBLE		0.72102	0.74859	0.67307	0.94292
JOIN (1)		0.77944	0.88187	0.79655	0.92650
JOIN (2)		0.72102	0.77365	0.79200	0.90262
JOIN (3)		0.72102	0.75484	0.79200	0.86857
JOIN (4)		0.72102	0.75484	0.75765	0.86861
JOIN (5)		0.72102	0.71136	0.67307	0.73745
					
MSVM-RFE (bootstrap)	5	0.95821	0.97247	0.92423	0.97401
	10	0.95821	**0.97851**	0.92288	**0.97525**
	15	0.95947	0.97457	0.92288	0.97401
	20	0.95947	0.97705	0.92315	0.97401
					
MSVM-RFE (boost)	5	0.95821	0.97247	0.92314	0.97401
	10	0.95821	0.97616	**0.92426**	0.97401
	15	0.95947	0.97247	0.92314	0.97401
	20	0.95947	0.97387	0.92314	0.97401

Numbers in parenthesis stands for cutoff value for JOIN method.

**Table 6:** Comparison of methods by maximum Az value using 8 features (Calcification)

	***T***	**MGH**	**WU**	**WFUSM**	**SHH**
SVM		0.91182	0.98765	0.94690	0.96595
SVM-RFE		**0.91196**	**1.00000**	0.95100	0.96595
					
ENSEMBLE		0.53915	0.69512	0.56583	0.91392
JOIN (1)		0.67508	0.71655	0.83947	0.93422
JOIN (2)		0.57971	0.72941	0.76157	0.88733
JOIN (3)		0.57971	0.72941	0.62686	0.73542
JOIN (4)		0.54571	0.69512	0.62686	0.72464
JOIN (5)		0.54571	0.69512	0.54077	0.66210
					
MSVM-RFE (bootstrap)	5	0.91182	0.98765	**0.95326**	**0.97868**
	10	0.91182	0.98765	0.95168	0.96595
	15	0.91182	0.98765	0.94690	0.96757
	20	0.91182	0.98765	0.94690	0.97348
					
MSVM-RFE (boost)	5	0.91182	0.98765	0.94690	0.96595
	10	0.91182	0.99259	0.94690	0.96595
	15	0.91182	0.99429	0.94690	0.96595
	20	0.91182	0.98765	0.94690	0.96595

Numbers in parenthesis stands for cutoff value for JOIN method.

**Table 7:** Comparison of methods by maximum Az value using 22 features (Mass)

	***T***	**MGH**	**WU**	**WFUSM**	**SHH**
SVM		0.88805	0.93642	0.92474	0.94998
SVM-RFE		0.88849	0.94173	0.93037	0.94998
					
ENSEMBLE		0.81490	0.90299	0.80317	0.86155
JOIN (1)		0.86728	0.92278	0.87638	0.90789
JOIN (2)		0.83034	0.93886	0.89597	0.85132
JOIN (3)		0.75098	0.87312	0.82694	0.83834
JOIN (4)		0.74270	0.74262	0.66948	0.83834
JOIN (5)		0.68776	0.71316	0.66948	0.80802
					
MSVM-RFE (bootstrap)	5	0.89720	0.93729	0.92664	0.95087
	10	0.88833	0.93666	0.92972	0.95016
	15	**0.89920**	0.93746	0.93000	0.95076
	20	0.89014	0.94290	0.92986	0.95066
					
MSVM-RFE (boost)	5	0.88993	0.93987	**0.93581**	0.94998
	10	0.88805	**0.94315**	0.92812	0.94998
	15	0.89092	0.94204	0.92789	0.94998
	20	0.88805	0.94197	0.92758	**0.95245**

Numbers in parenthesis stands for cutoff value for JOIN method.

**Table 8:** Comparison of methods by maximum Az value using 22 features (Calcification)

	***T***	**MGH**	**WU**	**WFUSM**	**SHH**
SVM		0.77497	0.91710	0.89738	0.94945
SVM-RFE		0.77497	**0.93436**	0.89859	0.95332
					
ENSEMBLE		0.68951	0.76647	0.72650	0.85677
JOIN (1)		0.75259	0.92326	0.81433	0.91352
JOIN (2)		0.72296	0.82307	0.72987	0.80400
JOIN (3)		0.70815	0.76647	0.70059	0.67598
JOIN (4)		0.58656	0.69779	0.65667	0.55964
JOIN (5)		0.53520	0.63858	0.65667	0.51203
					
MSVM-RFE (bootstrap)	5	0.77497	0.91710	0.89988	**0.95379**
	10	**0.77826**	0.91710	0.89786	0.95330
	15	0.77497	0.92193	0.89738	0.95250
	20	0.77497	0.93305	**0.90507**	0.95267
					
MSVM-RFE (boost)	5	0.77727	0.92097	0.89848	0.94945
	10	0.77497	0.93063	0.90108	0.95292
	15	0.77497	0.92352	0.90133	0.95136
	20	0.77497	0.92105	0.89957	0.95256

Numbers in parenthesis stands for cutoff value for JOIN method.

## Conclusion

In this paper, a new SVM-RFE based feature selection method was proposed. We conducted experiments on real world clinical data, and compared our method with baseline and other feature selection methods using SVM-RFE. Results show that our method outperforms in some cases and is at least competitive to others in other cases. Therefore, it can be a possible alternative to SVM-RFE or the original MSVM-RFE. Future works include investigation of specific methods to effectively combine models trained during the feature selection process and ways to combine feature subsets generated from individual SVM-RFE instances.

## Competing interests

The authors declare that they have no competing interests.

## Authors' contributions

SY carried out the study, designed and implemented the algorithms, conducted experiments and drafted this manuscript. SK supervised and instructed all research progress, and participated in the algorithm design and critical analysis of results. Both authors read and approved the final manuscript.
